# Evaluating the transferability of 15 European-derived fasting plasma glucose SNPs in Mexican children and adolescents

**DOI:** 10.1038/srep36202

**Published:** 2016-10-26

**Authors:** Christine Langlois, Arkan Abadi, Jesus Peralta-Romero, Akram Alyass, Fernando Suarez, Jaime Gomez-Zamudio, Ana I. Burguete-Garcia, Fereshteh T. Yazdi, Miguel Cruz, David Meyre

**Affiliations:** 1 Department of Clinical Epidemiology and Biostatistics, McMaster University, Hamilton, ON, Canada; 2 Medical Research Unit in Biochemistry, Hospital de Especialidades, Centro Médico Nacional Siglo XXI del Instituto Mexicano del Seguro Social, Mexico City, Mexico; 3 Centro de investigación sobre enfermedades infecciosas. Instituto Nacional de Salud Pública. Cuernavaca, Morelos, Mexico; 4 Department of Pathology and Molecular Medicine, McMaster University, Hamilton, ON, Canada

## Abstract

Genome wide association studies (GWAS) have identified single-nucleotide polymorphisms (SNPs) that are associated with fasting plasma glucose (FPG) in adult European populations. The contribution of these SNPs to FPG in non-Europeans and children is unclear. We studied the association of 15 GWAS SNPs and a genotype score (GS) with FPG and 7 metabolic traits in 1,421 Mexican children and adolescents from Mexico City. Genotyping of the 15 SNPs was performed using TaqMan Open Array. We used multivariate linear regression models adjusted for age, sex, body mass index standard deviation score, and recruitment center. We identified significant associations between 3 SNPs (*G6PC2* (rs560887), *GCKR* (rs1260326), *MTNR1B* (rs10830963)), the GS and FPG level. The FPG risk alleles of 11 out of the 15 SNPs (73.3%) displayed significant or non-significant beta values for FPG directionally consistent with those reported in adult European GWAS. The risk allele frequencies for 11 of 15 (73.3%) SNPs differed significantly in Mexican children and adolescents compared to European adults from the 1000G Project, but no significant enrichment in FPG risk alleles was observed in the Mexican population. Our data support a partial transferability of European GWAS FPG association signals in children and adolescents from the admixed Mexican population.

In 2011, 366 million people worldwide were diagnosed with diabetes, of which more than 90% were Type 2 Diabetes (T2D), and the prevalence may reach 552 million people by 2030^1^. T2D can be diagnosed based on one of several criteria and clinical symptoms of diabetes include polyuria, polydipsia, weight loss, blurred vision and fatigue^2^. Random blood glucose, oral glucose tolerance or glycosylated haemoglobin are also commonly used to diagnose T2D. However, fasting plasma glucose (FPG) is the most widely used biochemical tool to diagnose T2D in clinical setting (provisional diabetes diagnosis if FPG ≥ 7.0 mmol/L). FPG values predict incident cardiovascular outcomes in normoglycaemic and dysglycaemic subjects and is therefore an important biological marker in prevention of T2D and its complications^3^. Understanding the underlying physiology of FPG regulation is essential to improving our knowledge of T2D pathophysiology. Variations in FPG could stem from either genetic or environmental factors. Heritability estimates for FPG range from 38% to 51%, based on findings in twin studies^4^,^5^. Since 2008, GWAS have identified 53 FPG-associated loci mostly in European adult populations^6^,^7^. Nine of these FPG-associated loci were shown to interact with body mass index (BMI)^6^,^8^. Only a partial overlap was observed between GWAS loci associated with FPG level and T2D^9^. This supports the view that the genetic dissection of both extremes of the disease and intermediary quantitative traits is necessary for the understanding of glucose homeostasis^9^. FPG-associated loci identified in European populations were in part replicated in various ethnic groups^10^,^11^,^12^. Only one study to date have assessed the contribution of GWAS identified European adult FPG-associated loci in a youth population^13^. In this study, Barker *et al*. showed that 9 GWAS identified loci were associated with FPG levels in over 6,000 European children and adolescents with effect sizes comparable to adults^13^. However, the contribution of these GWAS identified loci to FPG level in non-European children has not been investigated so far.

The high obesity and dysglycemia predispositions of specific ethnic groups may be related to their past evolutive history^14^. James Neel suggested that the ‘thrifty’ genes would have been advantageous in the past, because it would allow for accumulation of fat quickly during times of abundance. This thrifty genotype would then increase individual survival during times of food scarcity. However, in modern societies with a constant abundance of food, this genotype efficiently prepares individuals for a hypothetical famine and predisposes to obesity and dysglycemia^14^. The Mexican population may be illustrative of this paradigm, as the prevalence of obesity and T2D are among the highest in the world^15^. In addition, the unique history of the Mexican population led to a complex admixed population, combining genomes of European, Native South-American and African ancestry with divergent susceptibility to metabolic diseases^16^.

Here we assessed the contribution of 15 loci initially associated with FPG in European adults in a population of 1,421 Mexican children and adolescents from Mexico City.

## Results

### Characteristics of the Mexican children population

The main anthropometric and biological characteristics of the 1,421 Mexican children are summarized in Table 1. Of the 1,421 Mexican children, 34 (2.4%) had FPG ≥ 5.6 mmol/L and 1 (0.07%) had FPG ≥ 7.0 mmol/L. In addition, 223 (15.8%) children presented clinical measures that were consistent metabolic syndrome as defined by the International Diabetes Federation (IDF) consensus of 2007^17^.

### Association of SNPs with fasting plasma glucose in Mexican children

Of the 15 SNPs tested, *MTNR1B* rs10830963 (β = 0.11 ± 0.04, *P* = 0.0091), *G6PC2* rs560887 (β = 0.12 ± 0.06, *P* = 0.049), and *GCKR* rs1260326 (β = 0.07 ± 0.04, *P* = 0.049) were significantly associated with FPG levels in Mexican children (Table 2) in a consistent direction of effect as those seen in initial GWAS reports in European adult populations (See Supplementary Table S2). The FPG risk alleles of a majority of SNPs (11 out of the 15 SNPs, 73.3%) had beta values for FPG directionally consistent with those reported in adult European GWAS. The genotype score (GS) was significantly associated with FPG in our population of Mexican children (β = 0.03 ± 0.01, *P* = 0.012).

### Association between genetic variant of fasting plasma glucose and continuous metabolic traits

The associations between the 15 genetic variants and 7 continuous metabolic traits are reported in Supplementary Table S1. Seven SNPs displayed a nominal evidence of association: *DGKB/TMEM195* rs2191349 with standard deviation score (SDS) BMI (β = 0.085 ± 0.038, *P* = 0.025) and with SDS-waist-to-hip ratio (SDS-WHR) (β = 0.044 ± 0.016, *P* = 0.008); *ADCY5* rs11708067 with SDS-WHR (β = −0.035 ± 0.016, *P* = 0.034); *FADS1* rs174550 with triglyceride (TG) (β = −0.085 ± 0.042, *P* = 0.045), with total cholesterol (Total-c) (β = 0.093 ± 0.039, *P* = 0.017) and LDL-cholesterol (LDL-c) (β = 0.145 ± 0.041, *P* = 0.00048); *GCKR* rs1260326 with TG (β = −0.092 ± 0.039, *P* = 0.018) and Total-c (β = −0.108 ± 0.003, *P* = 0.003); *GLIS3* rs7034200 with TG (β = −0.075 ± 0.036, *P* = 0.035); *MTNR1B* rs10830963 with HDL-cholesterol (HDL-c) (β = −0.109 ± 0.042, *P* = 0.010); and *SLC30A8* with fasting plasma insulin (FPI) level (β = 0.96 ± 0.044, *P* = 0.027). The GS showed a nominal association with lower TG (β = −0.024 ± 0.011, *P* = 0.035) and higher FPI levels (β = 0.026 ± 0.012, *P* = 0.033). After appropriate Bonferroni correction, no association of SNPs with metabolic traits remained significant (*P* < 4.5 × 10^−4^).

### Gene by gene interaction

We examined gene x gene interactions by testing pairwise interactions between the 15 SNPs on FPG (all possible combinations). While some interactions appeared nominally significant none survived Bonferroni correction. Interestingly, the SNP rs11708067 in *ADCY5* displayed nominal evidence of interaction with SNPs in/near 4 different genes (*FADS1, G6PC2, DGKB/TMEM195, GLIS3*) (See Supplementary Table S3).

### Allele frequency

Allele frequencies of the SNPs in this cohort were compared with those from European adults in the 1000 Genomes Project (1000G). The risk allele frequencies (RAFs) for 11 of 15 (73.3%) SNPs differed significantly in Mexican children compared to Europeans *(P* < 3.3 × 10^−3^) (Table 3). Of these, 6 were enriched and 5 were depleted in Mexican children and adolescents compared to Europeans (binomial test *P* = 1). Allele frequencies were also compared between Mexican children and Mexican adults from the 1000G reference panel (See Supplementary Table S4). The RAF of 1 SNP out of 15 was significantly enriched in Mexican children and adolescents compared to Mexican adults.

## Discussion

The growing rates of obesity, pre-diabetes and T2D are becoming a major health concern in the Mexican population^18^. The high lifetime risk of developing diabetes among Hispanic maybe explained by lifestyle and biological risk factors, including genetic susceptibility^19^,^20^. This study is the first to assess the transferability of previously identified European FPG SNPs in a population of Mexican children and adolescents. Our data support a partial transferability of FPG association signals in the admixed Mexican population. Three out of the 15 selected FPG SNPs (*G6PC2* rs560887*, GCKR* rs1260326 and *MTNRB1* rs10830963) were significantly associated with FPG in Mexican children. These SNPs are among the strongest genetic contributors previously identified in GWAS of adult populations of European ancestry^21^,^22^,^23^,^24^. Overall, the direction of effect of three-quarter of the FPG SNPs investigated is consistent with what was previously reported in GWAS^21^,^24^. This is consistent with data in children and adolescents of European ancestry where 56% of SNPs originally associated with FPG in adults replicated^13^. In addition, *G6PC2* rs560887 and *MTNRB1* rs10830963 SNPs are associated with FPG both in Mexican children and in European children and adolescents^13^. The GS also show a significant association with elevated FPG in Mexican children. Altogether, our data support a partial transferability of adult European FPG SNPs in Mexican children and adolescents. This observation is consistent with the fact that part of the Mexican genome is from European descent^25^,^26^. While ethnic-specific linkage disequilibrium structure may contribute to between population heterogeneity, it is likely to play a smaller role as the direction of effect of risk alleles is consistent with that in European adults for a majority of the SNPs. Alternatively, gene x gene, gene x lifestyle, gene x age interactions or epigenetic differences may contribute to between population heterogeneity^27^,^28^. Stressing the fact that FPG levels are not influenced by the same factors in children and adults is also important^29^. Our findings confirm that SNPs previously identified in specific ethnic groups are relevant candidates for association analyses in other populations^11^,^12^,^30^,^31^,^32^.

The association between the FPG SNPs and other metabolic traits was also investigated. These included SDS-BMI, SDS-WHR, TG, total-c, HDL-c, LDL-c and FPI. To ensure that the associations detected were not the result of indirect effects mediated by an increase in FPG, the models were adjusted accordingly. The results indicate a nominally significant association between the C-allele rs1260326 of *GCKR* and LDL-c, total-c and TG. This is consistent with the inverse association observed between rs1260326, lipids and FPG in European adults^33^. Similarly, the nominally significant association of *FADS1* rs174550 with TG and LDL-c has been previously reported in adult populations of European descent^21^,^34^. Thus our results, if confirmed, may suggest that the pleiotropic associations initially described in European adults are also transferable to Mexican children and adolescents.

When we examined the allele distribution between Mexican children and adolescents and European adults from 1000G, three-quarter of the 15 FPG SNPs tested displayed significant differences in the RAFs. There was no evidence of FPG increasing allele enrichment in Mexican children (binomial test, *P* = 1.00). If confirmed, the differences of RAF observed between European and Mexican populations most likely result from the unique history of the admixed Mexican population rather than local natural selection pressures. Overall, no major differences were observed for the FPG effect alleles between Mexican children and adolescents and Mexican adults from the 1000G. These data may suggest that the FPG SNPs have no major effect on longevity^35^.

We investigated gene x gene interactions and *ADCY5* showed a nominal interaction with 4 other gene variants in/near *DGKB/TMEM195, FADS1, G6PC2* and *GLIS3*. The adenylate cyclase 5 enzyme regulates the increase of Ca^2+^ in response to increased blood glucose levels. ADCY5 catalyzes the formation of the signaling molecule cAMP in response to G-protein signaling and mediates signaling downstream of the adrenergic receptor beta 1. Interestingly, we found that ADCY5 may potentially interact directly with at least 10 proteins, including several well-established FPG loci such as MTNR1B or GIPR (http://genomics.senescence.info/genes/human.html). The ability of ADCY5 to interact with other proteins may explain why gene x gene interactions at different loci are observed^36^. This is in agreement with the quantitative genetics model of fluxes and metabolic pools^37^. Inactivation of ADCY5 in mice impacts multiple pleiotropic traits as diverse as oxidative stress, energy balance, bone and cardiovascular health and longevity^36^,^38^,^39^. Furthermore, SNPs in *ADCY5* have shown robust associations with various traits such as birth weight, FPG, 2-hour glucose post OGTT and T2D^21^,^40^,^41^,^42^,^43^. Altogether, these data suggest that ADCY5 may be a key regulator of metabolism, even though the mechanisms underlying possible effect at the level of pancreatic β-cells remain unclear^44^. Although none of the genes that showed nominal interactions with *ADCY5* survived the Bonferroni adjustment, possibly due to our modest sample size (Supplementary Figure S2), further investigation of these genes could lead to new insight on the interactions between FPG loci in Mexican and other populations.

Our study has several strengths. We assessed for the first time the transferability of FPG SNPs previously identified in European adults in a population of Mexican children and adolescents. The recruitment of Mexican children was limited to one city, which restricts the range of environmental exposures and increases the power to identify genetic associations with multifactorial traits. By testing the possibility of gene pleiotropy, gene epistasis or local natural selection signatures, our study goes beyond traditional post-GWAS replication studies. However, our study is not without its caveats. We acknowledge that our sample had a modest power to detect main genetic effects and gene x gene interactions (Supplementary Figure S2). Our list of SNPs (N = 15) is not the most up to date as 45 SNPs have been conclusively associated with FPG in European adult populations so far. We did not adjust for population substructure within our sample, which may increase the risk of false positive association^45^. The cross-sectional nature of this study precludes causal inferences to be made about the associations described here.

In summary, our findings suggest a partial transferability of FPG SNPs identified in European adult populations in the admixed population of Mexican children and adolescents. Our data confirm the high trans-ethnic replicability of GWAS results and the value of performing GWAS in diverse ethnic groups to elucidate the molecular underpinnings of dysglycemia^32^. Our results support that a subset of SNPs modulate FPG levels early in life^46^. This may help to design and implement early personalized prevention strategies against dysglycemia and its complications in the future^47^.

## Methods

### Study population

A total of 1,559 children between the ages of 5 and 17 were randomly selected to participate in a cross-sectional study from four areas in Mexico City at the Primary Care Unit of the National Mexican Social Security Institute (Cuauhtémoc West, Independencia South, Nezahualcóyotl Est and Morelos North area). Recruitment was done in collaboration with local public schools. The study started in July 2011 and is still ongoing. A trained pediatrician performed all the anthropometric measurements. Blood samples were collected for biochemical measurements and DNA extraction. Children who had diagnosis of infectious disease, gastrointestinal disorders, administration of antimicrobial agents (within 6 months previous to study), incomplete questionnaires or biological samples were excluded. The child’s assent and written informed consent from the parents/guardians was obtained prior to enrolment into the study. The study protocol was approved by the Mexican Social Security Institute National Committee and the Ethical Committee Board. All procedures were conducted in accordance with the Declaration of Helsinki^48^.

### Anthropometric and Biochemical Measurements

Participants were scheduled for clinical laboratory evaluation following a 12 hour overnight fasting. All participants were weighed using a digital scale (Seca, Hamburg, Germany). Height was measured with a portable stadiometer (Seca 225, Hamburg, Germany). Height, weight and BMI, calculated as weight (kg)/height (m)^2^, were converted to age- and sex- adjusted standard deviation scores (SDS-Height, SDS-Weight and SDS-BMI, respectively) using the LMS method according to guidelines from the centers for disease control (CDC)^49^,^50^. Waist circumference (WC) and hip circumference (HC) were measured at the midpoint between the lowest rib and the iliac crest at the top of the iliac crest respectively, after a normal exhalation with children in the standing position. The WC and the waist to hip ratio (WHR) were also converted to age- and sex- adjusted standard deviation scores (SDS-WC and SDS-WHR, respectively) using the LMS method and growth charts based on US National Health and Nutrition Survey, cycle III (NHANES III)^51^. Systolic and diastolic blood pressure (SBP and DBP) were measured using a mercurial sphygmomanometer (ALPK2, Tokyo, Japan). Blood pressure readings were taken for each participant twice on the right arm in a sitting position with 5 minutes rest between each measurement and the mean of the two readings was determined. Age- and sex- adjusted standard deviations scores for SBP and DBP (SDS-SBP and SDS-DBP) were calculated using methods specified by the fourth report from the National High Blood Pressure Education Program (NHBPEP) in children and adolescents^52^. Blood samples were obtained following a 12 hour fast and were analyzed for FPG, total-c, HDL-c, LDL-c and triglycerides TG using the ILab 350 Clinical Chemistry System (Instrumentation Laboratory IL. Barcelona Spain). FPI (IU) was measured by chemiluminescence (IMMULITE, Siemens, USA). Metabolic syndrome was assigned based on the IDF consensus definition of the metabolic syndrome in children and adolescents^17^.

### Genotyping

Genomic DNA was extracted from peripheral blood with cells using the FLEX STAR Autogen platform (Holliston, Massachusetts US). One hundred twenty-eight SNPs that have been previously associated with metabolic traits were genotyped using the TaqMan OpenArray Real-Time PCR System (Life Technologies, Carlsbad, US), following the manufacturer’s instructions. Following quality control, 1,421 participants with both genotype and clinical data were retained for further analysis^53^. A list of SNPs that reached genome-wide significance (*P* < 5 × 10^−8^) with FPG level in adult populations of European ancestry was established. Three different strategies were used to optimize the SNP selection procedure using a key word search on i) the National Human Genome Research Institute (NHGRI) GWAS Catalog (www.genome.gov/gwastudies/) ii) the HuGE Navigator GWAS Integrator (www.hugenavigator.net/HuGENavigator/gWAHitStartPage.do) iii) the PubMed database (www.ncbi.nlm.nih.gov/pubmed). Using this strategy, 16 independent SNPs were selected in October 2012. One SNP was not successfully genotyped (rs11071657 near *C2CD4B*) whereas 15 SNPs passed the quality control criteria: rs560887 in *G6PC2*, rs1260326 in *GCKR*, rs4607517 near *GCK*, rs10830963 in *MTNR1B*, rs2191349 near *DGKB-TMEM195*, rs11708067 in *ADCY5*, rs7944584 in *MADD*, rs10885122 near *ADRA2A*, rs174550 in *FADS1*, rs11605924 in *CRY2*, rs11920090 in *SLC2A2,* rs7034200 in *GLIS3*, rs340874 near *PROX1*, rs13266634 in *SLC30A8,* rs7903146 in *TCF7L2*. We did not observe significant deviation from Hardy-Weinberg Equilibrium (HWE) (all *P* ≥ 0.0033) for the 15 SNPs and the average call rate was 97.59% for the 15 SNPs (See Supplementary Table S2). Individuals with greater than 10% missing genotypes were excluded from the analysis.

### Statistical Analyses

Allele frequencies in Europeans (N = 503) and Mexican adults (N = 64) were obtained from 1000G using Ensembl and compared to allele frequencies in Mexican children using chi-square tests as previously described^53^,^54^. Risk allele frequencies (RAF) were calculated using the FPG increasing allele reported in European GWAS (Table 3). Non-biological outlier data were discarded using a Cook’s distance test followed by an expert verification. Based on Shapiro-Wilk test (Supplementary Table S5), all the traits of interest deviated significantly from normality. Hence, rank based inverse normal transformations were applied wherever substantial deviations from normality were observed (See Supplementary Figure S1). Rank transformations corrected the lack of normality for all traits (Supplementary Table S5). Single SNP analyses were performed under the additive model, and the previously identified FPG increasing alleles for each of the 15 SNPs were used as the risk allele for the analyses. The association of SNPs/GS with FPG was assessed using linear regression models adjusted for age, sex, BMI and recruitment center. The association tests between SNPs/GS and additional metabolic traits were further adjusted for FPG to ensure that effects of SNPs were not the result of indirect associations with FPG (mediation *versus* pleiotropy). The genotype score (GS) was calculated by summing the alleles of the 15 FPG-associated SNPs so that the score ranged from 0 to 30. Since weighting has been shown to have no major impact on the overall GS^55^, an un-weighted GS was used for these analyses. We performed imputations for the missing genotypic values as previously described^56^. The imputation was performed for each locus using the mean number of the FPG alleles successfully genotyped for all individuals. For assessing gene x gene interactions, all possible pair-wise interactions between SNPs and FPG were tested (_*15*_*C*_*2*_ = 105) using linear regression models adjusted for age, sex, BMI and recruitment center. Two-tailed *P-*values are presented in this manuscript and *P* < 0.05 were considered significant when testing the association of the 15 SNPs and the GS with FPG in the Mexican children population, given the high prior probability of association. However, when testing those SNPs for HWE (*P* < 0.0033), differences of RAF between Mexican children and European adults from 1000G (*P* < 0.0033), and Mexican children and Mexican adults (*P* < 0.0033), gene x gene interactions (*P* < 4.8 × 10^−4^), and associations with metabolic traits (*P* < 4.5 × 10^−4^), correction for multiple testing was applied. We followed the strategy reported previously by Ronald J Feise and considered independent Bonferroni corrections for each question asked^57^. Statistical analyses were performed using R version 3.1.2. HWE was tested using the *HardyWeinberg* package^58^, LD was determined using *genetics* package, rank-based inverse-normal transformations were conducted using *GenABEL*^59^. Power calculations were performed using QUANTO (version 1.2.4, University of Southern California, Los Angeles, CA, USA).

## Additional Information

**How to cite this article**: Langlois, C. *et al*. Evaluating the transferability of 15 European-derived fasting plasma glucose SNPs in Mexican children and adolescents. *Sci. Rep.* **6**, 36202; doi: 10.1038/srep36202 (2016).

**Publisher’s note**: Springer Nature remains neutral with regard to jurisdictional claims in published maps and institutional affiliations.

## Supplementary Material

Supplementary Information

## Figures and Tables

**Table 1:** General characteristics of the studied population of Mexican children.

	N = 1421
Boys/Girls	755/667
Age (years)	9.25 (2.07)
Height (cm) [SDS-Height]	136.97 ± 13.61 [0.37 ± 1.02]
Weight (kg) [SDS-Weight]	38.04 ± 13.85 [0.8 ± 1.12]
BMI (kg/m^2^) [SDS-BMI]	19.67 ± 4.22 [0.81 ± 1.09]
LN/OW/OB	697/352/372 (49.1%/24.8%/26.2%)
WC (cm) [SDS-WC]	66.52 ± 11.82 [0.42 ± 1.09]
HC (cm)	78.26 ± 11.65
WHR [SDS-WHR]	0.85 ± 0.06 [2.95 ± 0.33]
SBP (mmHg) [SDS-SBP]	98.6 ± 11.16 [−0.3 ± 0.97]
DBP (mmHg) [SDS-DBP]	66.28 ± 8.94 [0.53 ± 0.77]
FPG (mmol/L)	4.57 ± 0.53
FPG > = 5.6 mmol/L	34 (2.4%)
FPG > = 7.0 mmol/L	1 (0.07%)
FPI (IU)	7.57 ± 7.65
TG (mg/dL)	93.79 ± 49.61
Total-c (mg/dL)	157.56 ± 33.41
HDL-c (mg/dL)	50.55 ± 12.78
LDL-c (mg/dL)	102.74 ± 26.36
Metabolic Syndrome	223 (15.8%)

Data are expressed as N (%) or mean ± standard deviation. Age- and sex- adjusted standard deviation scores (SDS) are enclosed by square brackets. Additional abbreviations: BMI, body mass index; WC, waist circumference; HC, hip circumference; WHR, waist-to-hip ratio; SBP, systolic blood pressure; DBP, diastolic blood pressure; FPG, fasting plasma glucose; FPI, fasting plasma insulin; TG, triglycerides; Total-c, total cholesterol; HDL-c, high-density lipoprotein cholesterol; and LDL-c, low-density lipoprotein cholesterol.

**Table 2:** Association of SNPs with FPG.

SNP	Gene	β ± SE	*P* = value
rs11708067	*ADCY5*	0.05 ± 0.04	0.156
rs10885122	*ADRA2A*	−0.02 ± 0.05	0.668
rs11605924	*CRY2*	0.03 ± 0.04	0.437
rs2191349	*DGKB/TMEM195*	0.02 ± 0.04	0.593
rs174550	*FADS1*	−0.08 ± 0.04	0.063
**rs560887**	***G6PC2***	**0.12 ± 0.06**	**0.049**
rs4607517	*GCK*	0.06 ± 0.05	0.186
**rs1260326**	***GCKR***	**0.07 ± 0.04**	**0.049**
rs7034200	*GLIS3*	−0.01 ± 0.03	0.85
rs7944584	*MADD*	0.1 ± 0.06	0.082
**rs10830963**	***MTNR1B***	**0.11 ± 0.04**	**0.009**
rs340874	*PROX1*	0 ± 0.04	0.942
rs11920090	*SLC2A2*	0.03 ± 0.06	0.620
rs13266634	*SLC30A8*	−0.03 ± 0.04	0.501
rs7903146	*TCL7L2*	0.03 ± 0.05	0.551
**GS**	**GS**	**0.03 ± 0.01**	**0.013**

The association of SNPs and the GS with FPG was determined using linear regression models adjusted for age, sex, recruitment center and BMI. Data presented are β ± SE, significant associations (*P* < 0.05) are indicated in bold.

**Table 3:** Risk allele frequency comparison between Mexican children and adult European from 1000G.

SNP	Gene	Risk Allele (A)	Other Allele (B)	Reference	Genotypes (AA/AB/BB)	Mexican RAF	European RAF	*P-*value	Status
rs11708067	*ADCY5*	A	G	Dupuis *et al*.^21^	547/654/213	0.618	0.829	2.6 × 10^−34^	Depleted
rs10885122	*ADRA2A*	G	T	Dupuis *et al*.^21^	1087/272/26	0.883	0.884	1.0	Equivalent
rs11605924	*CRY2*	A	C	Dupuis *et al*.^21^	285/725/402	0.459	0.506	1.1 × 10^−02^	Equivalent
rs2191349	*DGKB/ TMEM195*	T	G	Dupuis *et al*.^21^	245/669/478	0.416	0.505	1.4 × 10^−06^	Depleted
rs174550	*FADS1*	T	C	Dupuis *et al*.^21^	85/525/770	0.252	0.653	7.9 × 10^−114^	Depleted
rs560887	*G6PC2*	C	T	Bouatia-Naji *et al*.^23^	1161/232/6	0.913	0.701	1.7 × 10^−60^	Enriched
rs4607517	*GCK*	A	G	Prokopenko *et al*.^22^	43/444/923	0.188	0.176	4.3 × 10^−01^	Equivalent
rs1260326	*GCKR*	C	T	Vaxillaire *et al*.^33^	659/595/137	0.688	0.589	2.1 × 10^−08^	Enriched
rs7034200	*GLIS3*	A	C	Dupuis *et al*.^21^	483/617/262	0.581	0.521	1.1 × 10^−03^	Enriched
rs7944584	*MADD*	A	T	Dupuis *et al*.^21^	1117/253/12	0.9	0.726	1.9 × 10^−40^	Enriched
rs10830963	*MTNR1B*	G	C	Prokopenko *et al*.^22^	76/469/857	0.221	0.288	2.5 × 10^−05^	Depleted
rs340874	*PROX1*	C	T	Dupuis *et al*.^21^	180/629/568	0.359	0.531	3.2 × 10^−21^	Depleted
rs11920090	*SLC2A2*	T	A	Dupuis *et al*.^21^	1147/241/11	0.906	0.865	3.2 × 10^−04^	Enriched
rs13266634	*SLC30A8*	C	T	Dupuis *et al*.^21^	746/574/91	0.732	0.717	3.7 × 10^−01^	Equivalent
rs7903146	*TCF7L2*	C	T	Manning *et al*.^8^	991/341/34	0.85	0.683	3.5 × 10^−30^	Enriched

The RAF between Mexican children and European adults were compared using Χ^2^ tests. The RAFs for European adults were extracted from the 1000G project. Significantly (*P* < 0.0033) higher or lower RAFs in Mexican children compared to European adults are labeled as ‘Enriched’ or ‘Depleted’, respectively. Abbreviations: RAF, risk allele frequency.
