# Supplementary material for: Evaluating the transferability of 15 European-derived fasting plasma glucose SNPs in Mexican children and adolescents
Source: Sci Rep. 2016 Oct 26;6:36202. doi: 10.1038/srep36202 (PMC5080582; doi:10.1038/srep36202)
Supplement: Supplementary Information [file srep36202-s1.docx]

**Evaluating the transferability of 15 European-derived fasting plasma glucose SNPs in Mexican children and adolescents**

**Christine Langlois^1,#^, Arkan Abadi^1,#^, Jesus Peralta-Romero^2^, Akram Alyass^1^, Fernando Suarez^2^, Jaime Gomez-Zamudio^2^, Ana I. Burguete-Garcia^3^, Fereshteh T. Yazdi^1^, Miguel Cruz^2,*^, David Meyre^1,4,*^**

^1^Department of Clinical Epidemiology and Biostatistics, McMaster University, Hamilton, ON, Canada; ^2^Medical Research Unit in Biochemistry, Hospital de Especialidades, Centro Médico Nacional Siglo XXI del Instituto Mexicano del Seguro Social, Mexico City, Mexico; ^3^Centro de investigación sobre enfermedades infecciosas. Instituto Nacional de Salud Pública. Cuernavaca, Morelos, Mexico; ^4^Department of Pathology and Molecular Medicine, McMaster University, Hamilton, ON, Canada; **^#^**Shared first authorship.

**Corresponding author*:** Dr. David Meyre. Department of Clinical Epidemiology and Biostatistics, McMaster University, Room 3205, Michael DeGroote Centre for Learning & Discovery, 1280 Main Street West, Hamilton, ON L8S 4K1, Canada. Tel: 905.525.9140 Ext. 26802. Fax: 905.528.2814. Email: [meyred@mcmaster.ca](mailto:meyred@mcmaster.ca). Dr. Miguel Cruz, Unidad de Investigación Médica en Bioquímica, Hospital de Especialidades Centro Médico Nacional Siglo XXI, Instituto Mexicano del Seguro Social, Av. Cuauhtémoc, 330 C.P. 06725, México, D.F. Tel: 52 55 57612358; Fax: 5255 56276914. Email: [mcruzl@yahoo.com](mailto:mcruzl@yahoo.com).

| **Trait**  **SNP** | **SDS-BMI** | **SDS-WHR** | **TG** | **Total-c** | **LDL-c** | **HDL-c** | **FPI** |
| --- | --- | --- | --- | --- | --- | --- | --- |
| ***ADCY5* rs11708067** | β = -0.055 (0.038) *P =* 0.151 | β = -0.035 (0.016)  *P =* 0.034 | β = -0.059 (0.037)  *P =* 0.11 | β = -0.008 (0.034)  *P =* 0.82 | β = 0.015 (0.36)  *P =* 0.67 | β = -0.019 (0.036)  *P =* 0.59 | β = -0.021 (0.039)  *P =* 0.59 |
| ***ADRA2* rs10885122** | β = -0.011 (0.058) *P =* 0.85 | β = 0.015 (0.025)  *P =* 0.557 | β = 0.067 (0.055)  *P =* 0.23 | β = -0.033 (0.051)  *P =* 0.52 | β = -0.017 (0.54)  *P =* 0.76 | β = -0.083 (0.054)  *P =* 0.13 | β = 0.074 (0.059)  *P =* 0.21 |
| ***CRY2* rs11605924** | β = 0.010 (0.038)  *P =* 0.80 | β = 0.015 (0.017)  *P =* 0.374 | β = -0.017 (0.037)  *P =* 0.65 | β = -0.016 (0.034)  *P =* 0.64 | β = -0.041 (0.036)  *P =* 0.25 | β = -0.014 (0.036)  *P =* 0.70 | β = -0.047 (0.039)  *P =* 0.23 |
| ***DGKB.TMEM195* rs2191349** | β = 0.085 (0.038)  *P =* 0.025 | β= 0.044 (0.016)  *P =* 0.008 | β = 0.038 (0,037)  *P =* 0.30 | β = -0.010 (0.034)  *P =* 0.761 | β = -0.0005 (0.036)  *P =* 0.90 | β = 0.020 (0.036)  *P =* 0.58 | β = 0.059 (0.038)  *P =* 0.12 |
| ***FADS1* rs174550** | β = 0.011 (0.044)  *P =* 0.81 | β = 0.003 (0.019)  *P =* 0.857 | β = -0.085 (0.042)  *P =* 0.044 | β = 0.93 (0.039)  *P =* 0.017 | β = 0.145 (0.041)  *P =* 0.0005 | β = 0.029 (0.042)  *P =* 0.49 | β = -0.00003 (0.045)  *P =* 0.99 |
| ***G6PC2* rs560887** | β = -0.082 (0.068)  *P =* 0.226 | β = -0.021 (0.029)  *P =* 0.478 | β = 0.017 (0.066)  *P =* 0.800 | β = 0.012 (0.061) *P =* 0.84 | β = -0.016 (0.065)  *P =* 0.81 | β = 0.011 (0.065)  *P =* 0.87 | β = -0.065 (0.070)  *P =* 0.35 |
| ***GCK* rs4607517** | β = -0.088 (0.049)  *P =* 0.07 | β = -0.019 (0.021)  *P =* 0.377 | β = -0.085 (0.047)  *P =* 0.069 | β = -0.060 (0.043) *P =* 0.17 | β = -0.053 (0.046)  *P =* 0.25 | β = -0.021 (0.046)  *P =* 0.64 | β = -0.079 (0.050)  *P =* 0.11 |
| ***GCKR* rs1260326** | β = 0.027 (0.041)  *P =* 0.504 | β = 0.016 (0.018)  *P =* 0.378 | β = -0.092 (0.039)  *P =* 0.018 | β = -0.108 (0.003) *P =* 0.003 | β =-0.092 (0.038)  *P =* 0.015 | β = -0.047 (0.039)  *P =* 0.23 | β = 0.10 (0.041)  *P =* 0.14 |
| ***GLIS3* rs7034200** | β = -0.034 (0.037)  *P =* 0.36 | β = -0.007 (0.016)  *P =* 0.655 | β = -0.075 (0.036)  *P =* 0.035 | β = -0.022 (0.033) *P =* 0.50 | β = -0.016 (0.035)  *P =* 0.65 | β = 0.009 0.036  *P =* 0.80 | β = 0.029 (0.038)  *P =* 0.45 |
| ***MADD* rs7944584** | β = -0.057 (0.063)  *P =* 0.366 | β = 0.020 (0.028)  *P =* 0.472 | β = -0.023 (0.061)  *P =* 0.706 | β = -0.104 (0.056) *P =* 0.07 | β = -0.093 (0.06)  *P =* 0.12 | β = -0.043 (0.061)  *P =* 0.48 | β = 0.005 (0.066)  *P =* 0.94 |
| ***MTNR1B* rs10830963** | β = -0.016 (0.044)  *P =* 0.725 | β = 0.0003 (0.020)  *P =* 0.988 | β = 0.020 (0.043)  *P =* 0.638 | β = -0.029 (0.040)  *P =* 0.46 | β = -0.032 (0.042) *P =* 0.44 | β = -0.109 (0.04)  *P =* 0.01 | β = 0.081 (0.045)  *P =* 0.07 |
| ***PROX1* rs340874** | β = -0.018 (0.039)  *P =* 0.649 | β = 0.020 (0.017)  *P =* 0.229 | β = -0.012 (0.038)  *P =* 0.758 | β = -0.002 (0.035)  *P =* 0.95 | β = 0.021 (0.037) *P =* 0.58 | β = -0.039 (0.037)  *P =* 0.29 | β = 0.040 (0.040)  *P =* 0.32 |
| ***SLC2A2* rs11920090** | β = 0.085 (0.065)  *P =* 0.190 | β = 0.027 (0.028)  *P =* 0.328 | β = 0.023 (0.062)  *P =* 0.714 | β = 0.020 (0.057)  *P =* 0.74 | β = 0.069 (0.061) *P =* 0.25 | β = -0.043 (0.062)  *P =* 0.49 | β = -0.029 (0.067)  *P =* 0.66 |
| ***SLC30A8* rs13266634** | β = 0.022 (0.043)  *P =* 0.611 | β = 0.003 (0.019)  *P =* 0.891 | β = 0.004 (0.041)  *P =* 0.914 | β = 0.013 (0.038)  *P =* 0.74 | β = 0.006 (0.041) *P =* 0.49 | β = 0.010 (0.041)  *P =* 0.80 | β= 0.96  (0.044) P=0.027 0 |
| ***TCF7L2* rs7903146** | β = 0.004 (0.053)  *P =* 0.94 | β = 0.001 (0.023)  *P =* 0.962 | β = 0.045 (0.051)  *P =* 0.375 | β = -0.006 (0.047)  *P =* 0.895 | β = -0.034 (0.050) *P =* 0.49 | β = 0.020 (0.050)  *P =* 0.70 | β = 0.044 (0.053)  *P =* 0.41 |
| **Gene Score** | β = -0.005 (0.012)  *P =* 0.69 | β = 0.006 (0.005)  *P =* 0.271 | β = -0.024 (0.011)  *P =* 0.03 | β = -0.017 (0.010)  *P =* 0.11 | β = -0.009 (0.011) *P =* 0.44 | β = -0.019 (0.011)  *P =* 0.09 | β = 0.026 (0.012)  *P =* 0.03 |

**Supplementary Table S1.** Association between 15 fasting plasma glucose-associated SNPs, the genotype score and 7 continuous metabolic trait**s.** The association of SNPs with rank-transformed metabolic outcomes was determined using linear regression models adjusted for age, sex, recruitment center, and FPG level. Body mass index (BMI) and the waist-to-hip ratio (WHR) were first converted to age- and sex- adjusted standard deviations scores (SDS). The metabolic outcomes include; SDS-BMI, SDS-WHR, triglycerides (TG), total cholesterol (Total-c, mg/dL), HDL cholesterol (HDL-c, mg/dL), LDL cholesterol (LDL-c, mg/dL) and fasting plasma insulin (FPI, IU).

| **SNP** | **Gene** | **Chromosomal**  **position^a^** | **Major/Minor allele in Europeans** | **Fasting plasma glucose RAF in Europeans** | **Reference** | **Genotype count** | **Fasting plasma glucose risk allele frequency (%)** | **Genotype**  **call rate (%)** | **HWE**  ***P-*value** |
| --- | --- | --- | --- | --- | --- | --- | --- | --- | --- |
|  |  |  |  |  |  |  |  |  |  |
| **rs11708067** | ***ADCY5*** | chr3:123065778 | A/G | **A** | Dupuis et al. Nat Genet 2010 | 547/654/213 | 61.8 | 99.4 | 0.464 |
| **rs10885122** | ***ADRA2A*** | chr10:113042093 | G/T | **G** | Dupuis et al. Nat Genet 2010 | 1087/272/26 | 88.3 | 97.4 | 0.069 |
| **rs11605924** | ***CRY2*** | chr11:45873091 | C/A | **A** | Dupuis et al. Nat Genet 2010 | 402/725/285 | 45.9 | 99.3 | 0.218 |
| **rs2191349** | ***DGKB/ TMEM195*** | chr7:15064309 | T/G | **T** | Dupuis et al. Nat Genet 2010 | 478/669/245 | 41.6 | 97.9 | 0.699 |
| **rs174550** | ***FADS1*** | chr11:61571478 | T/C | **T** | Dupuis et al. Nat Genet 2010 | 770/525/85 | 25.2 | 97.0 | 0.775 |
| **rs560887** | ***G6PC2*** | chr2:169763148 | G/A | **G** | Bouatia-Naji et al. Science 2008 | 1161/232/6 | 91.3 | 98.4 | 0.132 |
| **rs4607517** | ***GCK*** | chr7:44235668 | G/A | **A** | Prokopenko et al. Nat Genet 2008 | 923/444/43 | 18.8 | 99.2 | 0.257 |
| **rs1260326** | ***GCKR*** | chr2:27508073 | C/T | **C** | Vaxillaire et al. Diabetes 2008 | 659/595/137 | 68.8 | 97.8 | 0.901 |
| **rs7034200** | ***GLIS3*** | chr9:4289050 | A/C | **A** | Dupuis et al. Nat Genet 2010 | 483/617/262 | 58.1 | 95.8 | 0.010 |
| **rs7944584** | ***MADD*** | chr11:47336320 | A/T | **A** | Dupuis et al. Nat Genet 2010 | 1117/253/12 | 90 | 97.2 | 0.656 |
| **rs10830963** | ***MTNR1B*** | chr11:92708710 | C/G | **G** | Prokopenko et al. Nat Genet 2008 | 857/469/76 | 22.1 | 98.6 | 0.278 |
| **rs340874** | ***PROX1*** | chr1:214159256 | C/T | **C** | Dupuis et al. Nat Genet 2010 | 581/629/185 | 35.9 | 96.8 | 0.770 |
| **rs11920090** | ***SLC2A2*** | chr3:170717521 | T/A | **T** | Dupuis et al. Nat Genet 2010 | 1147/241/11 | 90.6 | 98.4 | 0.875 |
| **rs13266634** | ***SLC30A8*** | chr8:118184783 | C/T | **C** | Dupuis et al. Nat Genet 2010 | 746/574/91 | 73.2 | 99.2 | 0.175 |
| **rs7903146** | ***TCL7L2*** | Chr10:112998590 | C/T | **C** | Manning et al. Nat Genet 2012 | 991/341/34 | 85 | 96.1 | 0.457 |

^a^NCBI Human Genome Browser (Fev. 2009 GRCh37/hg19) (dbSNP 138)

**Supplementary Table S2.** Description of 15 fasting plasma glucose-associated SNPs previously described in European adult populations. Chromosomal position was annotated according to the NCBI Human Genome Browser. The call rate (%) at each SNP is indicated as is the *P-*value from Fisher exact tests for HWE.

| **SNP 1** | **SNP 2** | **SNP 1 β** | **SNP 1 S.E** | **SNP 1 P-value** | **SNP 2 β** | **SNP 2 S.E** | **SNP 2 *P*.value** | **Interaction β** | **Interaction S.E** | **Interaction *P*-value** |
| --- | --- | --- | --- | --- | --- | --- | --- | --- | --- | --- |
| *ADCY5* (rs11708067) | *DGKB.TMEM195* (rs2191349) | 0.168 | 0.055 | 0.002 | 0.205 | 0.072 | 0.004 | -0.151 | 0.051 | 0.003 |
| *ADCY5* (rs11708067) | *FADS1* (rs174550) | -0.027 | 0.047 | 0.564 | -0.261 | 0.086 | 0.002 | 0.149 | 0.060 | 0.014 |
| *ADCY5* (rs11708067) | *G6PC2*  (rs560887) | 0.445 | 0.173 | 0.010 | 0.393 | 0.133 | 0.003 | -0.216 | 0.092 | 0.019 |
| *ADCY5* (rs11708067) | *GLIS3* (rs7034200) | -0.068 | 0.069 | 0.321 | -0.145 | 0.072 | 0.045 | 0.111 | 0.050 | 0.027 |
| *DGKB.TMEM195* (rs2191349) | *G6PC2*  (rs560887) | 0.497 | 0.176 | 0.005 | 0.336 | 0.103 | 0.001 | -0.257 | 0.094 | 0.006 |
| *FADS1* (rs174550) | *MADD* (rs7944584) | 0.277 | 0.161 | 0.085 | 0.248 | 0.098 | 0.011 | -0.195 | 0.088 | 0.027 |
| *G6PC2*  (rs560887) | *PROX1* (rs340874) | 0.280 | 0.092 | 0.002 | 0.462 | 0.181 | 0.011 | -0.248 | 0.097 | 0.010 |
| *GCKR* (rs1260326) | *SLC30A8* (rs13266634) | 0.255 | 0.098 | 0.009 | 0.143 | 0.095 | 0.131 | -0.124 | 0.062 | 0.044 |
| *MADD* (rs7944584) | *PROX1* (rs340874) | 0.237 | 0.089 | 0.008 | 0.302 | 0.159 | 0.058 | -0.175 | 0.086 | 0.042 |

**Supplementary Table S3.** Testing gene x gene interactions. All possible pairwise interactions between SNPs (*_15_C_2_*=105 unique combinations) were investigated using regression models that included a separate term for each SNP main effect as well as SNP by SNP interaction term. Only the nominally significant (*P* < 0.05) associations are shown. Note that none of these associations survived the Bonferroni-adjusted threshold of *P* < 4.8x10^-4^.

| **SNP** | **Gene** | **Risk**  **Allele**  **(A)** | **Other**  **Allele**  **(B)** | **Reference** | **Genotypes**  **(AA/AB/BB)** | **Mexican Children**  **RAF** | **Mexican Adults**  **RAF** | ***P*value** | **Status** |
| --- | --- | --- | --- | --- | --- | --- | --- | --- | --- |
| rs11708067 | *ADCY5* | A | G | Dupuis et al Nat Genet 2010 | 547/654/213 | 0.618 | 0.633 | 8.1 x10^-01^ | Equivalent |
| rs10885122 | *ADRA2A* | G | T | Dupuis et al Nat Genet 2010 | 1087/272/26 | 0.883 | 0.859 | 5.0 x10^-01^ | Equivalent |
| rs11605924 | *CRY2* | A | C | Dupuis et al Nat Genet 2010 | 285/725/402 | 0.459 | 0.500 | 4.1 x10^-01^ | Equivalent |
| rs2191349 | *DGKB.TMEM195* | T | G | Dupuis et al Nat Genet 2010 | 245/669/478 | 0.416 | 0.430 | 8.3 x10^-01^ | Equivalent |
| rs174550 | *FADS1* | T | C | Dupuis et al Nat Genet 2010 | 85/525/770 | 0.252 | 0.312 | 1.5 x10^-01^ | Equivalent |
| rs560887 | *G6PC2* | C | T | Bouatia-Naji et al Science 2008 | 1161/232/6 | 0.913 | 0.859 | 5.6 x10^-02^ | Equivalent |
| rs4607517 | *GCK* | A | G | Prokopenko et al Nat Genet 2008 | 43/444/923 | 0.188 | 0.148 | 3.1 x10^-01^ | Equivalent |
| rs1260326 | *GCKR* | C | T | Vaxillaire et al. Diabetes 2008 | 659/595/137 | 0.688 | 0.648 | 4.0 x10^-01^ | Equivalent |
| rs7034200 | *GLIS3* | A | C | Dupuis et al Nat Genet 2010 | 483/617/262 | 0.581 | 0.602 | 7.1 x10^-01^ | Equivalent |
| rs7944584 | *MADD* | A | T | Dupuis et al Nat Genet 2010 | 1117/253/12 | 0.900 | 0.820 | 6.0 x10^-03^ | Equivalent |
| rs10830963 | *MTNR1B* | G | C | Prokopenko et al Nat Genet 2008 | 76/469/857 | 0.221 | 0.195 | 5.6 x10^-01^ | Equivalent |
| rs340874 | *PROX1* | C | T | Dupuis et al Nat Genet 2010 | 180/629/568 | 0.359 | 0.438 | 8.7 x10^-02^ | Equivalent |
| rs11920090 | *SLC2A2* | T | A | Dupuis et al Nat Genet 2010 | 1147/241/11 | 0.906 | 0.820 | 2.0 x10^-03^ | Enriched |
| rs13266634 | *SLC30A8* | C | T | Dupuis et al Nat Genet 2010 | 746/574/91 | 0.732 | 0.758 | 5.9 x10^-01^ | Equivalent |
| rs7903146 | *TCF7L2* | C | T | Manning et al Nat Genet 2012 | 991/341/34 | 0.850 | 0.781 | 4.6 x10^-02^ | Equivalent |

**Supplementary Table S4. Risk allele frequency comparison between Mexican children and adolescents and Mexican adults from 1000G.** The RAF between Mexican children and adolescents and Mexican adults were compared using Chi-square tests. The RAF in Mexican adults was extracted from the 1000G project. Significantly higher or lower RAFs in Mexican children and adolescents compared to Mexican adults are labeled as ‘Enriched’ or ‘Depleted’, respectively. Abbreviations: RAF, risk allele frequency.

|  | **Shapiro-Wilk Test (*P*-value)** | |
| --- | --- | --- |
| **Trait** | **Untransformed** | **Transformed** |
| SDS-BMI | 7.42 x 10^-16^ | 1.000 |
| SDS-WHR | 1.62 x 10^-08^ | 1.000 |
| Triglycerides (mg/dL) | 7.75 x 10^-36^ | 1.000 |
| Total cholesterol (mg/dL) | 7.08 x 10^-09^ | 1.000 |
| HDL cholesterol (mg/dL) | 2.26 x 10^-08^ | 0.907 |
| LDL cholesterol (mg/dL) | 2.36 x 10^-13^ | 1.000 |
| Fasting plasma insulin (IU) | 4.28 x 10^-36^ | 1.000 |
| Fasting plasma glucose (mmol/L) | 4.20 x 10^-08^ | 0.564 |

**Supplementary Table S5.** Shapiro Wilk tests of normality. Shapiro Wilk tests were performed on metabolic outcomes to determine whether they are normally distributed. All of the traits showed significant (*P* < 0.05) departures from normality. Shapiro Wilk tests were also performed after these metabolic traits were rank transformed, and the *P*-values reflect the effectiveness of normalization using rank transformation. Body mass index (BMI) and the waist-to-hip ratio (WHR) were first converted to age- and sex- adjusted standard deviations scores (SDS-BMI and SDS-WHR respectively).

**
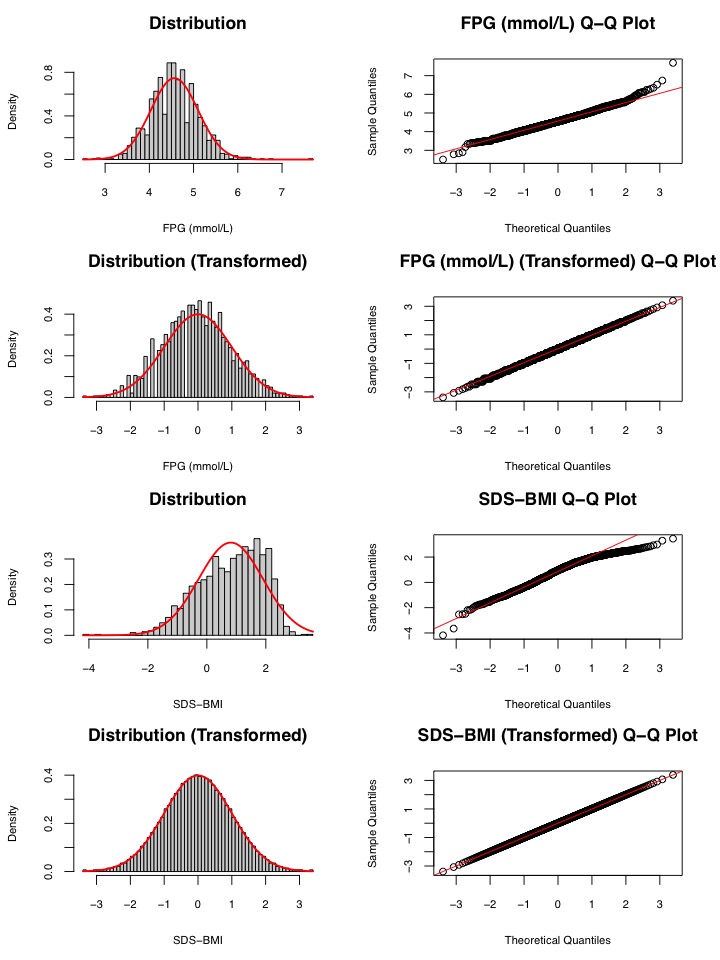
**

**
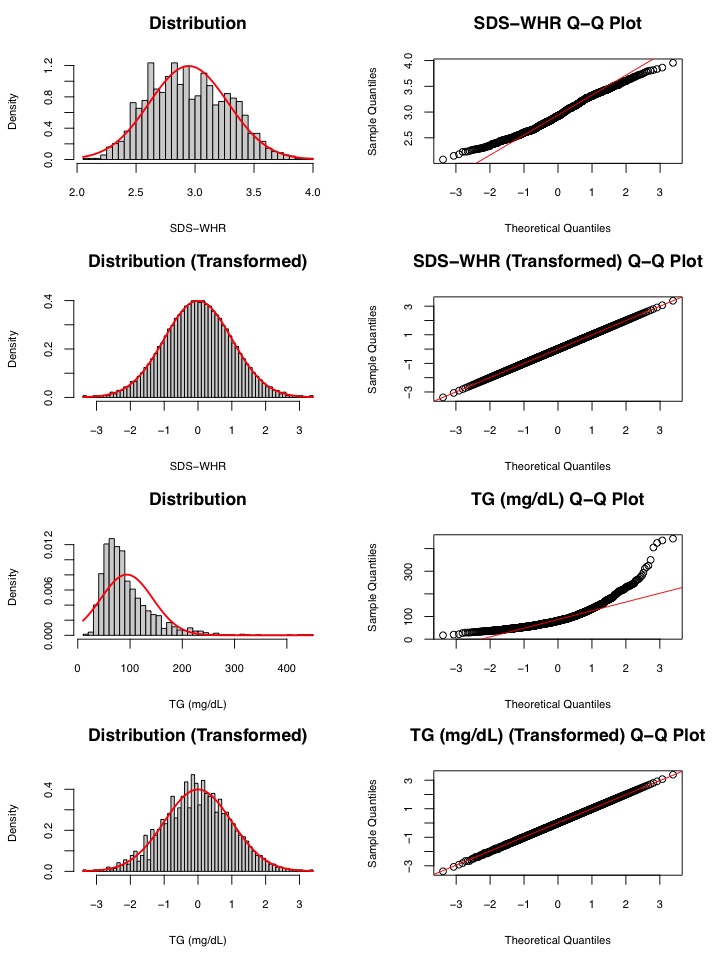
**

**
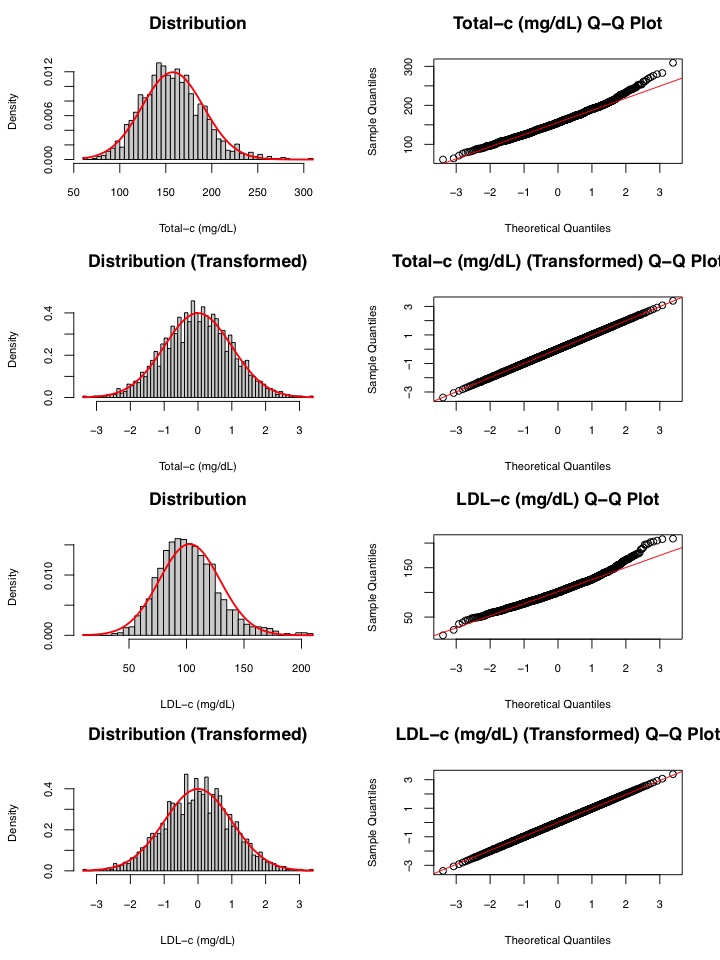
**

**
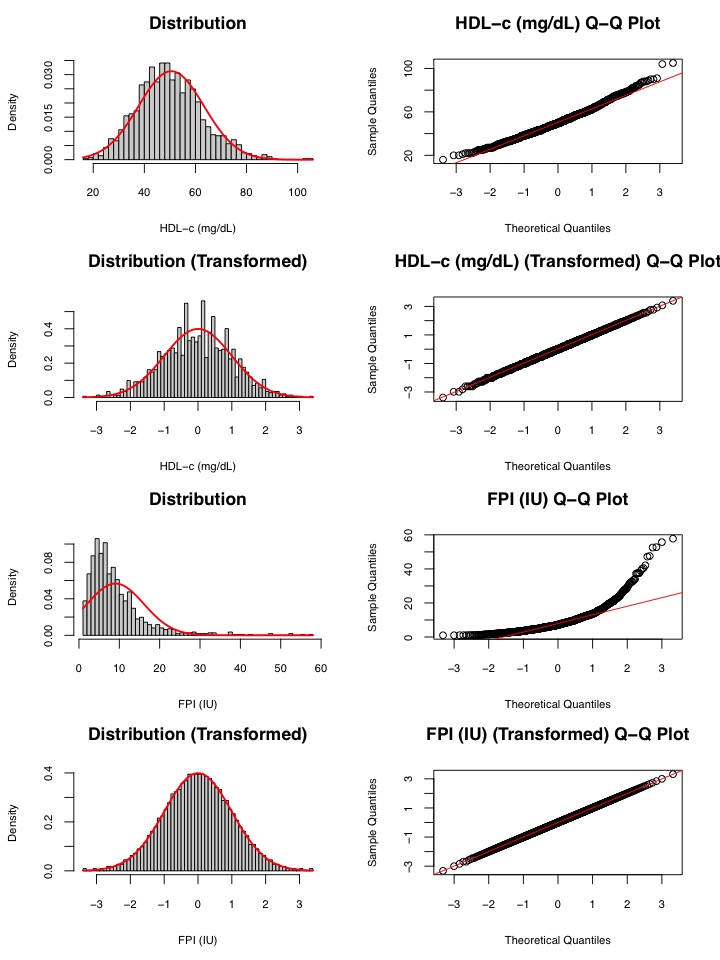
**

**Supplementary Figure S1:** The distributions of metabolic outcomes before and after rank transformation are presented. Each of these is accompanied by corresponding Q-Q plots. Body mass index (BMI) and the waist-to-hip ratio (WHR) were first converted to age- and sex- adjusted standard deviations scores (SDS). The metabolic outcomes include; SDS-BMI, SDS-WHR, triglycerides (TG, mg/dL), total cholesterol (Total-c, mg/dL), HDLcholesterol (HDL-c, mg/dL), LDL cholesterol (LDL-c, mg/dL), fasting plasma insulin (FPI, IU), and fasting plasma glucose (FPG, mmol/L).

**Supplementary figure S2a:** Power calculation for main genetic effect of SNPs on fasting plasma glucose for a 2-sided *P*-value = 0.05. The sample size required to detect the indicated effects with 80% power is indicated.

**Supplementary figure S2b:** Power calculation for gene x gene interaction effects on fasting plasma glucose for a 2-sided *P*-value=0.05. The sample size required to detect the indicated effects with 80% power is indicated.

**Supplementary figure S2c:** Power calculation for gene x gene interaction effects on fasting plasma glucose with adjustment for multiple testing for a 2-sided *P*-value=0.00014. The sample size required to detect the indicated effects with 80% power is indicated.

.
